# Supplementary material for: End Sequence Analysis Toolkit (ESAT) expands the extractable information from single-cell RNA-seq data
Source: Genome Res. 2016 Oct;26(10):1397–410. doi: 10.1101/gr.207902.116 (PMC5052061; doi:10.1101/gr.207902.116)
Supplement: Supplemental Material [file supp_gr.207902.116_Supp_Materials.tar.gz › ESAT-esat_v0.1/doc/html/classbroad_1_1pda_1_1seq_1_1rap_1_1rna_1_1_transcriptome_command_line_program-members.html]

ESAT: Member List


ESAT


- Main Page
- Related Pages
- Packages
- Classes
- Files

- Class List
- Class Index
- Class Hierarchy
- Class Members

All Classes Namespaces Files Functions Variables Enumerator Pages

- broad
- pda
- seq
- rap
- rna
- TranscriptomeCommandLineProgram

broad.pda.seq.rap.rna.TranscriptomeCommandLineProgram Member List

This is the complete list of members for broad.pda.seq.rap.rna.TranscriptomeCommandLineProgram, including all inherited members.

|  |  |  |
| --- | --- | --- |
| ANNOTATION | broad.pda.seq.rap.GenomeCommandLineProgram |  |
| COORD\_SPACE | broad.pda.seq.rap.GenomeCommandLineProgram |  |
| coordinateSpace | broad.pda.seq.rap.GenomeCommandLineProgram | protected |
| customCommandLineValidation() | broad.pda.seq.rap.rna.TranscriptomeCommandLineProgram | protected |
| GENE | broad.pda.seq.rap.GenomeCommandLineProgram |  |
| GENE\_BED | broad.pda.seq.rap.rna.TranscriptomeCommandLineProgram |  |
| getCoordinateSpace() | broad.pda.seq.rap.GenomeCommandLineProgram | protected |
| getGenes() | broad.pda.seq.rap.GenomeCommandLineProgram |  |
| getGenomicSpace() | broad.pda.seq.rap.rna.TranscriptomeCommandLineProgram | protected |
| getRegions() | broad.pda.seq.rap.GenomeCommandLineProgram |  |
| getRegionSet() | broad.pda.seq.rap.GenomeCommandLineProgram |  |
| getRegionsMap() | broad.pda.seq.rap.GenomeCommandLineProgram |  |
| getTranscriptomeSpace() | broad.pda.seq.rap.rna.TranscriptomeCommandLineProgram | protected |
| isGenomicSpace() | broad.pda.seq.rap.GenomeCommandLineProgram |  |
| loadAlignmentModel(File bamFile, boolean pairedEnd) | broad.pda.seq.rap.rna.TranscriptomeCommandLineProgram |  |
| broad::pda::seq::rap::GenomeCommandLineProgram.loadAlignmentModel(File bamFile) | broad.pda.seq.rap.GenomeCommandLineProgram |  |
| loadCoordinateSpace() | broad.pda.seq.rap.rna.TranscriptomeCommandLineProgram | protected |
| loadRNAAlignmentModel(File bamFile, boolean pairedEnd) | broad.pda.seq.rap.rna.TranscriptomeCommandLineProgram |  |
| MASK\_FILE | broad.pda.seq.rap.GenomeCommandLineProgram |  |
| MAX\_FRAGMENT\_LENGTH | broad.pda.seq.rap.GenomeCommandLineProgram |  |
| MIN\_MAPPING\_QUALITY | broad.pda.seq.rap.GenomeCommandLineProgram |  |
| PCT\_MASKED\_ALLOWED | broad.pda.seq.rap.GenomeCommandLineProgram |  |
| REGION | broad.pda.seq.rap.GenomeCommandLineProgram |  |
| SIZES | broad.pda.seq.rap.GenomeCommandLineProgram |  |
| TRANSCRIPTION\_READ | broad.pda.seq.rap.rna.TranscriptomeCommandLineProgram |  |
| ts | broad.pda.seq.rap.rna.TranscriptomeCommandLineProgram | protected |


---

Generated on Fri Aug 15 2014 15:11:03 for ESAT by  

 1.8.7
